# The role of glyceraldehyde 3-phosphate dehydrogenase (GapA-1) in *Neisseria meningitidis *adherence to human cells

**DOI:** 10.1186/1471-2180-10-280

**Published:** 2010-11-09

**Authors:** Sarfraz A Tunio, Neil J Oldfield, Dlawer AA Ala'Aldeen, Karl G Wooldridge, David PJ Turner

**Affiliations:** 1 Molecular Bacteriology and Immunology Group, Centre for Biomolecular Sciences, University of Nottingham, Nottingham, NG7 2RD, UK

## Abstract

**Background:**

Glyceraldehyde 3-phosphate dehydrogenases (GAPDHs) are cytoplasmic glycolytic enzymes, which although lacking identifiable secretion signals, have also been found localized to the surface of several bacteria (and some eukaryotic organisms); where in some cases they have been shown to contribute to the colonization and invasion of host tissues. *Neisseria meningitidis *is an obligate human nasopharyngeal commensal which can cause life-threatening infections including septicaemia and meningitis. *N. meningitidis *has two genes, *gapA-1 *and *gapA-2*, encoding GAPDH enzymes. GapA-1 has previously been shown to be up-regulated on bacterial contact with host epithelial cells and is accessible to antibodies on the surface of capsule-permeabilized meningococcal cells. The aims of this study were: 1) to determine whether GapA-1 was expressed across different strains of *N. meningitidis*; 2) to determine whether GapA-1 surface accessibility to antibodies was dependant on the presence of capsule; 3) to determine whether GapA-1 can influence the interaction of meningococci and host cells, particularly in the key stages of adhesion and invasion.

**Results:**

In this study, expression of GapA-1 was shown to be well conserved across diverse isolates of *Neisseria *species. Flow cytometry confirmed that GapA-1 could be detected on the cell surface, but only in a *siaD*-knockout (capsule-deficient) background, suggesting that GapA-1 is inaccessible to antibody in *in vitro*-grown encapsulated meningococci. The role of GapA-1 in meningococcal pathogenesis was addressed by mutational analysis and functional complementation. Loss of GapA-1 did not affect the growth of the bacterium *in vitro*. However, a GapA-1 deficient mutant showed a significant reduction in adhesion to human epithelial and endothelial cells compared to the wild-type and complemented mutant. A similar reduction in adhesion levels was also apparent between a *siaD*-deficient meningococcal strain and an isogenic *siaD gapA-1 *double mutant.

**Conclusions:**

Our data demonstrates that meningococcal GapA-1 is a constitutively-expressed, highly-conserved surface-exposed protein which is antibody-accessible only in the absence of capsule. Mutation of GapA-1 does not affect the *in vitro *growth rate of *N. meningitidis*, but significantly affects the ability of the organism to adhere to human epithelial and endothelial cells in a capsule-independent process suggesting a role in the pathogenesis of meningococcal infection.

## Background

*Neisseria meningitidis *is an obligate human commensal that is spread from person to person by droplet infection. The organism colonizes the nasopharyngeal mucosa in an asymptomatic manner, a condition known as carriage [1]. Under certain circumstances the bacteria can invade the epithelial layers to gain access to the bloodstream, which can result in a wide spectrum of clinical syndromes ranging from transient bacteraemia to rapidly fatal sepsis. Bacteria may also interact with cerebrovascular endothelial cells and cross the blood-cerebrospinal fluid barrier to cause meningitis [2]. To reach the meninges, *N. meningitidis *must interact with two cellular barriers and adhesion to both epithelial and endothelial cells are crucial stages of infection. Adhesion to both cell types is complex and remains poorly understood, but initial attachment is mediated by type IV pili, which is followed by contact-dependent down-regulation of pili and capsule: structures that otherwise hinder intimate adhesion, in a process that may involve the CrgA protein [3]. Intimate interaction between bacterial membrane components and their respective host cell surface receptors may subsequently lead to uptake of the bacterial cells (reviewed in [4]).

Glyceraldehyde 3-phosphate dehydrogenase (GAPDH) is a glycolytic enzyme which catalyzes the conversion of glyceraldehyde 3-phosphate to 1, 3-diphosphoglycerate. The most common form is the NAD+-dependent enzyme (EC 1.2.1.12) found in all organisms studied so far and which is usually located in the cytoplasm. In addition to its metabolic function, studies have demonstrated that GAPDH is present on the surface of several microbial pathogens and may facilitate their colonization and invasion of host tissues by interacting directly with host soluble proteins and surface ligands. Surface localization of GAPDH was first demonstrated in the Gram-positive pathogen, *Streptococcus pyogenes*. In this organism, surface-exposed GAPDH binds several mammalian proteins including the uPAR/CD87 membrane protein on pharyngeal cells [5-8], regulates intracellular host cell signalling events [9] and contributes to host immune evasion [10]. GAPDH was subsequently identified on the surface of other Gram-positive bacteria including staphylococci [11,12], *S. agalactiae *[13], *S. pneumoniae *[14] and *Listeria monocytogenes *[15]. In addition, surface localization of GAPDH has been reported in enterohemorrhagic (EHEC) and enteropathogenic (EPEC) *Escherichia coli*; the protein of these pathogens has been observed to bind to human plasminogen and fibrinogen, suggesting a role in pathogenesis [16]. Similar to the surface-localized GAPDHs from other species, the EHEC and EPEC GAPDH proteins possess NAD-ribosylating activity [17]. In *Mycoplasma genitalium*, surface-associated GAPDH is important for adhesion to human mucin [18], and in *Lactobacillus plantarum*, a normal inhabitant of the human gastrointestinal tract, GAPDH was shown to be involved in adherence to gastric mucin and Caco-2 cells [19,20]. Interestingly, the major fimbriae of *Porphyromonas gingivalis *bind to GAPDH on the surface of several oral streptococci, and this interaction is important for colonization of the oral cavity [21]. Fungi also express GAPDH on their cell surface, for example, the GAPDH of *Candida albicans *was shown to be associated with the cell wall and involved in mediating adhesion to fibronectin, laminin and plasminogen [22-24]. GAPDH has also been found on the surface of the single-celled protozoan, *Trichomonas vaginalis*, and shown to bind extracellular matrix components, including fibronectin [25].

The *N. meningitidis *MC58 genome sequence contains two putative GAPDH-encoding genes (*gapA-1 *and *gapA-2*) which share 50% nucleotide identity [26]. Expression of GapA-1 (but not GapA-2) on the meningococcal cell surface was previously found to be up-regulated following contact with human epithelial cells, although no function was ascribed to this observation [27]. Two other cytoplasmic glycolytic enzymes, despite lacking identifiable secretion signals, anchoring motifs or hydrophobic membrane-spanning regions (hence the term 'anchorless proteins'), have been found localized to the surface of *N. meningitidis*. These are enolase, which acts to recruit plasminogen onto the bacterial surface [28], and fructose-1, 6-bisphosphate aldolase (FBA), which we have recently demonstrated is required for optimal adhesion to human cells [29]. The aim of this study was to determine whether GapA-1 can influence the interaction of meningococci and host cells.

## Methods

### Bacterial strains and growth conditions

*E. coli *TOP10F' and BL21(DE3)pLysS (Table 1) were used for the expression of 6 × histidine-tagged recombinant GapA-1 encoded by plasmid pDT-GapA1 (Table 1). *E. coli *JM109 was used as host for the construction of mutagenic and complementation plasmids, pSAT-8 and pSAT-14 respectively. *E. coli *strains were grown at 37°C in LB broth or on LB agar supplemented, where appropriate, with ampicillin (100 μg ml^-1^), kanamycin (30 μg ml^-1^) or erythromycin (200 μg ml^-1^). Strains of *Neisseria *(Table 1 and Additional file 1) were grown at 37°C, air plus 5% CO_2, _on Brain Heart Infusion (BHI) agar supplemented with 1% Vitox (Thermo Fisher Scientific, Waltham, MA) and kanamycin (50 μg ml^-1^) or erythromycin (5 μg ml^-1^) where appropriate.

**Table 1:** Bacterial strains and plasmids

Strain or plasmid	Description	Source or reference
Strains		
*E. coli*		
JM109	Cloning strain	Promega, Madison, WI
TOP10F'	Cloning strain	Invitrogen, Carlsbad, CA
BL21(DE3)pLysS	Expression strain	Invitrogen, Carlsbad, CA
*N. meningitidis*		
MC58	wild-type serogroup B strain	[26]
MC58Δ*gapA-1*	*gapA-1 *deletion and replacement with kanamycin cassette	This study
MC58Δ*gapA-1 gapA-1*^*Ect*^	MC58Δ*gapA-1 *complemented with an ectopic copy of *gapA-1*	This study
MC58Δ*siaD*	*siaD *deletion and replacement with erythromycin cassette	C. Tang Imperial College
MC58Δ*siaD *Δ*gapA-1*	*siaD *and *gapA-1 *deficient strain generated from MC58Δ*siaD *using pSAT-8	This study
Plasmids		
pCRT7/NT-TOPO	Cloning vector encoding resistance to ampicillin	Invitrogen, Carlsbad, CA
pDT-GapA1	MC58 *gapA-1 *gene cloned in pCRT7-TOPO	This study
pGEM-T Easy	Cloning vector encoding resistance to ampicillin	Promega, Madison, WI
pSAT-6	3-kb fragment spanning the MC58 *gapA-1 *region cloned in pGEM-T Easy	This study
pJMK30	Source of kanamycin resistance cassette	[43]
pSAT-8	pSAT-6 containing the kanamycin resistance cassette in the same orientation as the deleted *gapA-1 *gene	This study
pSAT-12	Complementation vector containing *cbbA *and encoding resistance to erythromycin	[29]
pSAT-14	pSAT-12 containing *gapA-1 *in place of the deleted *cbbA*	This study

### DNA manipulation

Genomic DNA was extracted from *N. meningitidis *using the DNeasy Tissue kit (Qiagen, Crawley, UK). Plasmid DNA was prepared using the QIAprep Spin kit (Qiagen, Crawley, UK). All enzymes were purchased from Roche Diagnostics (Indianapolis, IN) and used according to the manufacturer's instructions. DNA sequencing was carried out at the School of Biomedical Sciences (University of Nottingham) on an ABI 377 automated DNA sequencer.

### Preparation of recombinant GapA-1 and αGapA-1 rabbit polyclonal antiserum

The *gapA-1 *gene was amplified from *N. meningitidis *MC58 using oligonucleotide primers NMB0207(F) and NMB0207(R) (Table 2). The amplicon was ligated into pCRT7/NT-TOPO and the resulting plasmid, pDT-GapA1, used to transform *E. coli *BL21(DE3)pLysS. Transformants were grown to log phase, induced for 3 h with 1 mM isopropyl β-D-1-thiogalactopyranoside (IPTG) and harvested by centrifugation. Recombinant 6 × histidine-tagged GapA-1 was then affinity-purified under denaturing conditions. Briefly, the culture pellet was dissolved in 20 ml lysis buffer (100 mM NaH_2_PO_4_, 10 mM Tris-Cl, 10 mM Imidazole and 8 M Urea, pH 8.0) and disrupted by sonication using a MSE Soniprep 150 for 10 cycles (each cycle consisting of a 10 s burst followed by a 10 s cooling period). The cell lysates was then mixed with 1 ml HisPur™ Cobalt Resin (Thermo Fisher Scientific, Waltham, MA) and incubated overnight at 4°C. The lysate-resin mixture was then applied to the column, and washed with 100 mM NaH_2_PO_4_, 10 mM Tris-Cl, 20 mM Imidazole, 8 M Urea, pH 6.3. Bound proteins were then eluted in elution buffer (100 mM NaH_2_PO_4_, 10 mM Tris-Cl, and 8 M Urea, pH 4.5). Eluted fractions were resolved by SDS-PAGE, and recombinant GapA-1 excised from the gel, transferred to Mini D-Tube dialyzers (Merck Biosciences, Darmstadt, Germany) and electro-eluted according to the recommendations of the manufacturer. Recombinant GapA-1 was then concentrated using YM-30 Centrifugal filter units (Millipore, Billerica, MA). To generate rabbit antiserum against purified recombinant GapA-1, a New Zealand White female rabbit was immunized subcutaneously four times at 2-week intervals with 30 μg of protein emulsified in Freund's complete (first immunization only) or incomplete adjuvant.

**Table 2:** List of primers used in this study

Primer	DNA sequence*	Restriction site
Expression		
NMB0207(F)	CGC**GGATCC**ATGGGCATCAAAGTCGCCATC	*Bam*HI
NMB0207(R)	CGC**GTCGAC**TTATTTGAGCGGGCGCACTTC	*Sal*I
Mutagenesis		
NMB0207(R)FL	GAGAACTGTCATGCGTATTCC	
NMB0207(F)FL	CCAAACCCAATGCCGCGAATG	
gapA1_M1(IR)	GCG**AGATCT**GCAACAAACCGTC	*Bgl*II
gapA1_M2(IF)	GCG**AGATCT**GGTTTGTTCCTTTGTTGAGGG	*Bgl*II
Complementation		
pSAT-12iPCR(IF)	CGC**AGATCT**GATACCCCCGATGAC	*Bgl*II
pSAT-12iPCR(IR)	CGC**AGATCT**CATTTGTGTC TCCTTGG	*Bgl*II
gapA1_Comp(F)2	CGC**GGATCC**ATGGGCATCAAAGTC	*Bam*HI
gapA1_Comp(R)2	CGC**GGATCC**TTTGTTTGACGGTTTGTTG	*Bam*HI

*All primers were designed from the *N. meningitidis *MC58 genome sequence. Sequences in bold identify restriction enzyme sites.

### SDS-PAGE and immunoblotting

Proteins were electrophoretically separated using 10% polyacrylamide gels (Mini-Protean III; Bio-Rad, Hercules, CA) and were stained using SimplyBlue Safestain™ (Invitrogen, Carlsbad, CA) or transferred to nitrocellulose membranes as previously described [30]. Membranes were probed with mouse anti-pentahistidine antibody (Qiagen, Crawley, UK) or rabbit primary antibody diluted 1:10,000 & 1:1000 respectively in blocking buffer (5% [wt/vol] non fat dry milk, 0.1% [vol/vol] Tween 20 in 1 × phosphate-buffered saline [PBS]) and incubated for 2 h. After being washed in PBS with 0.1% Tween 20 (PBST), membranes were incubated for 2 h with 1:30,000-diluted goat anti-mouse (or anti-rabbit) IgG-alkaline phosphatase conjugate (Sigma-Aldrich, St. Louis, MI). After washing with PBST, blots were developed using BCIP/NBT-Blue liquid substrate (Sigma-Aldrich, St. Louis, MI).

### Construction of MC58Δ*gapA-1*

A *ca. *3 kb fragment of DNA consisting of the *gapA-1 *gene and flanking DNA was amplified using NMB0207(F)FL and NMB0207(R)FL (Table 2) from *N. meningitidis *MC58 chromosomal DNA. The amplified DNA was cloned into pGEM-T Easy to generate pSAT-6 (Table 1). This was then subject to inverse PCR using primers gapA1_M1(IR) and gapA1_M2(IF) (Table 2) resulting in the amplification of a 5 kb amplicon in which the *gapA-1 *coding sequence was deleted and a unique *Bgl*II site had been introduced. The *Bgl*II site was used to introduce a kanamycin resistance cassette from pJMK30 (Table 1) in place of *gapA-1*. One of the resulting plasmids, pSAT-8, containing the resistance cassette in the same orientation as the deleted gene, was confirmed by restriction digestion and sequencing and subsequently used to mutate meningococcal strains by natural transformation and allelic exchange as previously described [31]. Mutation of *gapA-1 *was confirmed by PCR analysis and immunoblotting.

### Complementation of *gapA-1*

Plasmid pSAT-12, which we previously used to complement the meningococcal *cbbA *gene [29] was subjected to inverse PCR using the primers pSAT-12iPCR(IF) and pSAT-12iPCR(IR) (Table 2). This resulted in deletion of the *cbbA *coding sequence but leaving the upstream *cbbA-*promoter sequence intact and introduced a unique *Bgl*II site to facilitate the cloning of *gapA-1 *downstream of the promoter. The *gapA-1 *coding sequence was amplified from strain MC58 using the primers gapA1_Comp(F)2 and gapA1_Comp(R)2 (Table 2) incorporating *Bam*HI-sites into the amplified fragment. The *Bam*HI-digested fragment was then introduced into the *Bgl*II site to yield pSAT-14. This vector therefore contained the *gapA-1 *coding sequence under the control of the *cbbA *promoter and downstream of this, an erythromycin resistance gene. These elements were flanked by the MC58 genes NMB0102 and NMB0103. pSAT-14 was then used to transform MC58Δ*gapA-1 *by natural transformation, thus introducing a single chromosomal copy of *gapA-1 *under the control of the *cbbA *promoter and the downstream erythromycin resistance cassette in the intergenic region between NMB0102 and NMB0103. Insertion of the *gapA-1 *gene and erythromycin resistance cassette at the ectopic site was confirmed by PCR analysis and sequencing.

### Flow cytometry

These experiments were performed essentially as previously described [29]. Briefly, 1 × 10^7 ^CFU aliquots of *N. meningitidis *were incubated for 2 h with rabbit anti-GapA-1-specific polyclonal antiserum (RαGapA-1) (1:500 diluted in PBS containing 0.1% BSA, 0.1% sodium azide and 2% foetal calf serum) and untreated cells were used as a control. Cells were washed with PBS and incubated for 2 h with goat anti-rabbit IgG-Alexa Fluor 488 conjugate (Invitrogen, Carlsbad, CA; diluted 1:50 in PBS containing 0.1% BSA, 0.1% sodium azide and 2% foetal calf serum). Again, untreated cells were used as a control. Finally, the samples were washed before being fixed in 1 ml PBS containing 0.5% formaldehyde. Samples were analyzed for fluorescence using a Coulter Altra Flow Cytometer. Cells were detected using forward and log-side scatter dot plots, and a gating region was set to exclude cell debris and aggregates of bacteria. A total of 50,000 bacteria (events) were analyzed.

### Association and invasion assays

Association and invasion assays were performed essentially as previously described [29]. Briefly, human brain microvascular endothelial (HBME) or HEp-2 cells were grown to confluence in 24-well tissue culture plates and infected with approximately 1 × 10^6 ^CFU of log-phase meningococci (multiplicity of infection of 10 bacteria per cell) and incubated for 2 h (association) or 4 h (invasion) in 5% CO_2 _at 37°C. To assess total cell association, monolayers were washed, then disrupted and homogenized in 1 ml 0.1% saponin in PBS. To assess invasion, monolayers were further incubated in DMEM containing gentamicin (100 μg ml^-1^) for 2 h. Prior to further steps, aliquots of the gentamicin-containing supernatants were plated out to confirm killing of extra-cellular bacteria. Furthermore, the susceptibility of all meningococcal strains to gentamicin at 100 μg ml^-1 ^was confirmed prior to testing. Monolayers were then washed, disrupted and homogenized in 1 ml 0.1% saponin in PBS. Meningococci were enumerated by serial dilution of the homogenized suspensions and subsequent determination of colony-forming units by plating aliquots from appropriate dilutions of the lysates on agar. All association and invasion assays were repeated at least three times. Statistical significance was measured using a two-tailed Student *t*-test.

### Protein and nucleic acid sequence analysis

Public databases containing previously published protein and DNA sequences were searched using the BLAST and PSI-BLAST programs available at http://blast.ncbi.nlm.nih.gov/Blast.cgi. The genome database of *N. meningitidis *MC58 was interrogated at http://cmr.jcvi.org/cgi-bin/CMR/GenomePage.cgi?org=gnm. Sequence homology data were obtained using the CLUSTALX software (http://www.clustal.org/). Protein secretion signals were analyzed using the SignalP 3.0 server available at http://www.cbs.dtu.dk/services/SignalP/[32]. GenBank accession numbers for the *gapA-1 *sequences analyzed in this study are as follows: YP_97432562 (FAM18), YP_00160027 (ST-4821 strain 053442), YP_002341615 (Z2491), YP_208807 (gonococcal strain FA1090) and ZP_03723143 (*N. lactamica *ATCC 23970).

## Results

### Sequence analysis of *gapA-1*, flanking DNA and GapA-1 protein

In *N. meningitidis *strain MC58, *gapA-1 *(locus tag NMB0207) is located downstream of, and in the opposite orientation to, *aat *(NMB0206) encoding the leucyl/phenylalanyl-tRNA-protein transferase and upstream of, and in the same orientation as, NMB0208, which encodes an electron transport protein, ferredoxin (4Fe-4S-type). A similar genomic arrangement is present in the meningococcal strains Z2491 [33], FAM18 [34] and 053442 [35]. The sequences of *gapA-1 *in these strains are >97% identical to the MC58 *gapA-1 *gene. Additionally, *gapA-1 *orthologues are found in the gonococcal strain FA1090 (99% identical) and *N. lactamica *strain ST640 (93% identical). At the amino acid level, the highly conserved GAPDH active site was identified (^153^ASCTTNCL^160^), and GapA-1 shows significant homology to GAPDH enzymes from higher organisms, including the human GAPDH enzyme (45% identity). Despite its demonstrated presence on the bacterial surface [27], GapA-1 of *N. meningitidis *was not predicted to be an exported protein by the SignalP-HMM and -NN programs.

### Cloning, expression and purification of recombinant GapA-1

**Figure 1:**
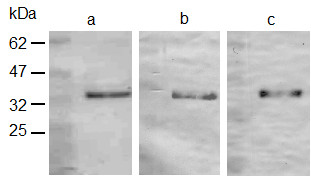
**SDS-PAGE and immunoblot analysis of recombinant GapA-1**. SDS-PAGE analysis confirms the purity of the recombinant GapA-1 purified under denaturing conditions (a). Immunoblot analysis shows that recombinant GapA-1 is recognized by RαGapA-1 (b) and anti-pentahistidine antibodies (c).

The *gapA-1 *gene from MC58 was cloned into the expression vector pCRT7/NT-TOPO to facilitate the expression and subsequent purification of 6 × histidine-tagged recombinant GapA-1 (Figure 1a). This was used to generate RαGapA-1. Immunoblot analysis confirmed that RαGapA-1 and anti-pentahistidine antibodies both reacted to the purified recombinant GapA-1 (Figure 1b &1c).

### Construction of an *N. meningitidis gapA-1 *null mutant strain

**Figure 2:**
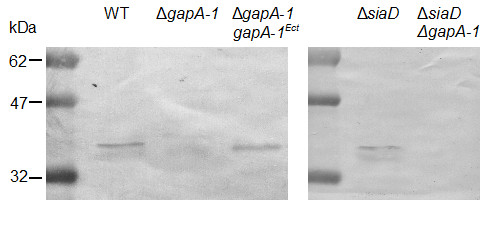
**Immunoblot analysis of whole cell proteins from *N. meningitidis *using RαGapA-1**. Analysis of MC58 wild-type, Δ*gapA-1 *mutant derivative and complemented mutant reveals the absence of GapA-1 in the Δ*gapA-1 *mutant preparation. Similar analysis shows the abolition of GapA-1 expression in the MC58Δ*siaD *Δ*gapA-1 *mutant compared to the parental MC58Δ*siaD *strain.

To examine the roles of GapA-1 in the meningococcus, a *gapA-1 *knockout derivative of *N. meningitidis *MC58 was generated. Immunoblotting using RαGapA-1 showed that GapA-1 could be detected in whole cell lysates of wild-type but not MC58Δ*gapA-1 *(Figure 2, lanes 1 & 2) confirming that GapA-1 was expressed under the conditions used and that expression had been abolished in the mutant. This analysis further confirmed that the RαGapA-1 sera did not recognize GapA-2 (37-kDa) under the conditions used. To further confirm that the immuno-reactive protein was GapA-1, a wild-type copy of *gapA-1 *was introduced *in trans *into MC58Δ*gapA-1 *using plasmid pSAT-14 (Table 1). Introduction of *gapA-1 *at an ectopic site restored GapA-1 expression (Figure 2, lane 3). Further immunoblot analyses using a panel of 14 *N. meningitidis *strains (Additional file 1) including representatives of differing serogroups and MLST-types showed that GapA-1 expression was conserved across all strains (data not shown). Expression was also conserved in *N. gonorrhoeae *FA1090 (data not shown). These data complement *in silico *predictions that GapA-1 is universally present and suggests that GapA-1 is constitutively-expressed across pathogenic *Neisseria *species.

### Meningococcal GapA-1 is only surface-accessible to antibodies in the absence of capsule

**Figure 3:**
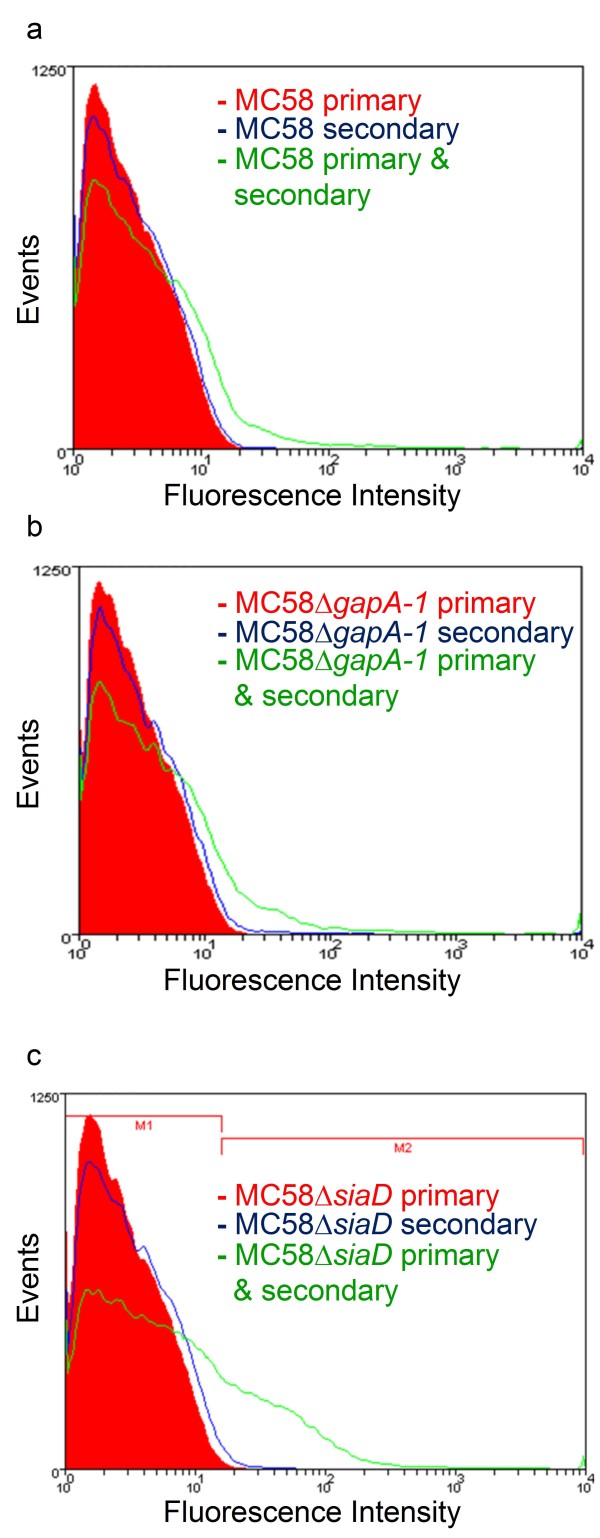
**Flow cytometry of MC58 wild-type (a), MC58Δ*gapA-1 *(b) or MC58Δ*siaD *(c) for GapA-1 surface localization**. Cells were stained with RαGapA-1 (primary alone), anti-rabbit IgG-Alexa Fluor 488 conjugate (secondary alone) or both. Fluorescence was displayed as a histogram. In panel c, the histogram area in M2 represents the population of fluorescently labelled meningococci.

Grifantini *et al *showed using flow cytometry that GapA-1 was accessible to specific antibodies on the surface of meningococci [27]. However, the methodology used involved pre-treatment of the cells with 70% ethanol to permeabilize the capsule, making it unclear whether GapA-1 was accessible to antibodies in encapsulated bacteria. We used RαGapA-1 antibodies to probe wild-type, MC58Δ*gapA-1 *and MC58Δ*siaD *(capsule-deficient) strains and analyzed them by flow cytometry (Figure 3). MC58 wild-type and MC58Δ*gapA-1 *treated with RαGapA-1 followed by anti-rabbit IgG-Alexa Fluor 488 conjugate showed no demonstrable shift in fluorescence signal compared to the same strains incubated with RαGapA-1 or secondary antibody alone showing that GapA-1 was not detectable on whole cells of these strains (Figure 3a &3b). However, identical experiments using MC58Δ*siaD *demonstrated a clear shift in fluorescence when cells were treated with RαGapA-1 followed by anti-rabbit IgG-Alexa Fluor 488 conjugate (Figure 3c). This demonstrated that, in the absence of capsule, surface exposed GapA-1 was accessible to antibody. From the MC58Δ*siaD *cells probed with both antibodies, 25% were found in the M2 region (Figure 3c), suggesting that in broth-grown cells unexposed to human epithelial cells only a minority of the population had GapA-1 was present on the cell surface. Pre-immune sera showed no reactivity against wild-type MC58 or MC58Δ*siaD*, and RαGapA-1 specifically recognized only GapA-1 in immunoblot experiments confirming that the binding of RαGapA-1 to MC58Δ*siaD *observed by flow cytometry was GapA-1 specific.

### GapA-1 is required for optimal adhesion to host cells

**Figure 4:**
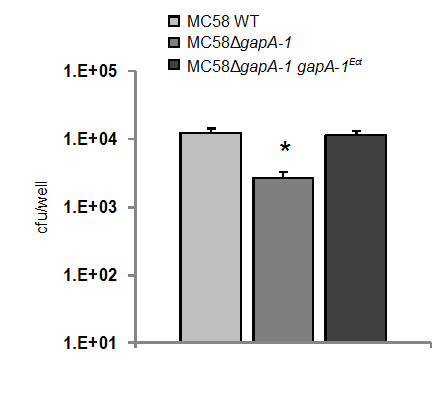
**MC58Δ*gapA-1 *has a reduced ability to associate with HBMEs compared to the wild-type or complemented strains**. The number of GapA-1-deficient meningococci associating was significantly lower than the wild-type (*P = 0.0018). Mean levels shown from three independent experiments, each using triplicate wells. Bars denote standard deviation. Cfu denotes colony forming units.

The capacity of the wild-type, GapA-1 mutant and complemented mutant strains to associate with, and invade into human brain microvascular endothelial (HBME) cells were then determined. GapA-1 deficient meningococci had a significantly reduced capacity to adhere to monolayers of HBME cells (Figure 4). No significant reduction was observed in the ability of the GapA-1 mutant to invade monolayers of HBME cells (data not shown). Similar results were also obtained using HEp-2 cells confirming that the effect was not limited to endothelial cells (data not shown). To confirm that the observed effects were not due to an impairment of *in vitro *growth, the growth rate of the strains was compared by measuring the optical density at 600 nm (OD_600_) and determining the viable counts of broth cultures sampled during exponential growth over 24 h in triplicate on three separate occasions. No significant difference between strains was observed (data not shown).

### The effect of GapA-1 mutation on meningococcal adhesion is capsule-independent

**Figure 5:**
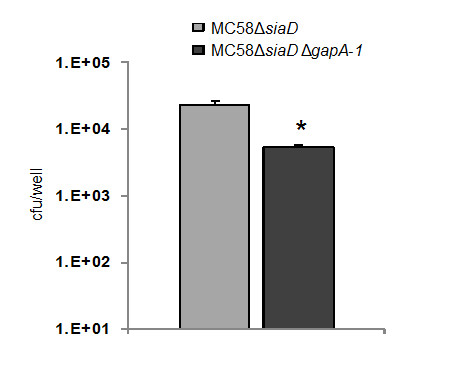
**MC58Δ*siaD *Δ*gapA-1 *has a reduced ability to associate with HBME cells compared to MC58Δ*siaD***. The number of MC58Δ*siaD *Δ*gapA-1 *cells associating was significantly lower than the capsule null (*P = 0.0008). Mean levels shown from three independent experiments, each using triplicate wells. Bars denote standard deviation. Cfu denotes colony forming units.

Our experiments demonstrate clearly that GapA-1 increased the ability of meningococci to adhere to host cells (Figure 4), suggesting a role in pathogenesis. However, flow cytometry indicated that GapA-1 is made inaccessible to antibodies on the surface of meningococci by capsule (Figure 3). In order to determine whether capsule expression influences the role of GapA-1 in adhesion to host cells we constructed a *gapA-1 *deficient derivative of MC58Δ*siaD*, which does not express a capsule. After confirming that GapA-1 expression had been abolished in MC58Δ*siaD *Δ*gapA-1 *(Figure 2, lanes 4 & 5), we determined the capacity of both strains to associate with HBME cells. GapA-1 deficient non-encapsulated meningococci had a significantly reduced capacity to adhere to monolayers of HBME cells compared to the parent strain (Figure 5), confirming our observation that GapA-1 is required for optimal host cell adhesion. However, this reduction was not enhanced in the non-encapsulated background, indicating that the role of *gapA-1 *in the adhesion process was not moderated by the production of capsule. In summary, these experiments show that GapA-1 plays a role in the adherence of *N. meningitidis *with human cells in a capsule-independent manner.

## Discussion

It is now apparent that many of the classical cytoplasmic house-keeping enzymes, including enolase, FBA and GAPDH, are often localized to the surface of microbial pathogens, where they exhibit various functions, unrelated to their housekeeping roles [36-38]. Currently, there is considerable interest in identifying the additional roles of these bacterial glycolytic enzymes. In *N. meningitidis*, enolase was recently shown to be a surface-localized protein, where it acts to recruit plasminogen onto the bacterial surface [28]. In addition, we have recently demonstrated that FBA is also a partially surface-localized protein and is required for optimal adhesion to human cells through an unknown mechanism [29]. Furthermore, it is noteworthy that GAPDH is also a multi-functional protein in eukaryotic cells. For example, in addition to its role in central metabolic pathways, GAPDH is involved in controlling cell survival by delaying apoptosis via the inhibition of caspase-dependant proteolysis [39]. This raises the possibility that GAPDH on the surface of invasive bacterial pathogens such as *N. meningitidis *may influence intracellular processes of host cells to the advantage of the invading organism (including delaying apoptosis).

In our study, attempts to purify GapA-1 under native conditions were unsuccessful. Therefore, recombinant GapA-1 was purified under denaturing conditions in order to raise antiserum, but it was not possible to determine whether this protein had enzymatic activity. Rabbit polyclonal GapA-1 antiserum was used for immunoblot analysis of whole cell proteins from different clinical isolates of known MLST-type. These strains were representatives from lineages commonly causing invasive meningococcal disease. This showed that they all express GapA-1 suggesting that GapA-1 is constitutively-expressed in *N. meningitidis*. A GapA-1 knock-out mutant was created in *N. meningitidis *strain MC58 to facilitate studies of the potential role of GapA-1 in the pathogenesis of meningococcal disease. The GapA-1 mutant grew at the same rate (in broth culture and on solid media) as the wild-type and the complemented mutant strains, demonstrating that GapA-1 is not required for growth of the meningococcus under *in vitro *conditions. No differences in either colony or bacterial cell morphology (using light microscopy) were observed.

In a previous study, Grifantini *et al. *used microarrays to show that expression of *gapA-*1 was up-regulated in meningococcal strain MC58 (4.8-fold) following contact for 30 min with human 16HBE14 epithelial cells [27]. Subsequent flow cytometry experiments showed that GapA-1 could be detected on the cell surface of free grown and adherent meningococci [27]. However, the methodology used involved a pre-treatment of cells with 70% ethanol to permeabilize the capsule layer, thus making it unclear if GapA-1 is antibody-accessible in encapsulated meningococci. In our study, GapA-1 could only be detected on the meningococcal cell surface in mutants lacking capsule, suggesting that GapA-1 is usually masked by this structure.

In our adhesion experiments using *siaD*-knockout meningococci, the GapA-1 mutant strain exhibited a similarly significantly reduced capacity to adhere to host cells compared to the GapA-1 mutant in an encapsulated strain suggesting that the presence of capsule does not affect the role of GapA-1 in the adhesion process. It is not obvious why the influence of GapA-1 on adhesion is not itself modulated by the presence of masking capsule since the removal of capsule does increase the ability of meningococci to bind host cells via outer membrane adhesins [4]. In our adhesion experiments the binding of strains lacking capsule was approximately two-fold higher than the cognate encapulsulated strains (Figure 4 &5). This agrees with previous studies comparing the adherence of encapsulated and non-capsulated serogroup B meningococci to macrophages and buccal epithelial cells, where four-fold and less than two-fold increases, respectively, in adhesion were seen when capsule production was abolished [40,41]. Thus, it is possible that the influence of surface-localised GapA-1 on adhesion to host cells is indirect, possibly involving its enzymatic activity, and that a direct interaction of GapA-1 with the host cell surface is not required. Alternatively, capsule expression in meningococci is known to be down-regulated following the initial type IV pilus dependant-contact with host cells in order to facilitate intimate adherence [3]. Thus, we hypothesize that surface-localised GapA-1 may be unmasked following this change allowing it to influence subsequent steps in adhesion.

The observation that GapA-1 is detectable on the meningococcal cell surface suggests that GapA-1 is actively translocated to the outer membrane. An alternative hypothesis is that GapA-1 is released from lysed cells and recruited back onto the surface of intact meningococci. This maybe unlikely given the recent work on *L. plantarum *which showed that provoked cell lysis did not lead to re-association of GAPDH onto the cell surface [42]. Instead, it was suggested that changes in plasma membrane permeability during the growth cycle may be involved in the movement of GAPDH onto the external surface of the plasma membrane in this Gram-positive organism [42]. Clearly, such a mechanism could only account for periplasmic localization in a Gram-negative organism. We are currently investigating how GapA-1 is localized to the cell surface in *N. meningitidis*.

## Conclusions

Meningococcal GapA-1 is a constitutively-expressed, highly-conserved surface-exposed protein which is antibody-accessible only in the absence of capsule. Mutation of GapA-1 does not affect the *in vitro *growth rate of *N. meningitidis*, but significantly affects the ability of the organism to adhere to human epithelial and endothelial cells in a capsule-independent process suggesting a role in the pathogenesis of meningococcal infection.

## Authors' contributions

SAT carried out experiments and was involved in manuscript editing. NJO performed experiments and wrote the majority of the manuscript. DAAA, KGW and DPJT participated in the design of the experiments and wrote and edited portions of the manuscript. All authors have read and approved the final manuscript.

## Supplementary Material

Additional file 1 **Isolates of *N. meningitidis *examined for the expression of GapA-1**. Click here for file

## References

[B1] Caugant DA Maiden MCJ Meningococcal carriage and disease - population biology and evolution Vaccine 2009 27 Suppl 2 B64 B70 10.1016/j.vaccine.2009.04.061 19464092 PMC2719693

[B2] Stephens DS Biology and pathogenesis of the evolutionarily successful, obligate human bacterium *Neisseria meningitidis* Vaccine 2009 27 Suppl 2 B71 77 10.1016/j.vaccine.2009.04.070 19477055 PMC2712446

[B3] Deghmane AE Giorgini D Larribe M Alonso JM Taha MK Down-regulation of pili and capsule of *Neisseria meningitidis *upon contact with epithelial cells is mediated by CrgA regulatory protein Mol Microbiol 2002 43 1555 1564 10.1046/j.1365-2958.2002.02838.x 11952904

[B4] Virji M Pathogenic neisseriae: surface modulation, pathogenesis and infection control Nature 2009 7 274 286 10.1038/nrmicro2097 19287450

[B5] Lottenberg R Broder CC Boyle MD Kain SJ Schroeder BL Curtiss R Cloning, sequence analysis, and expression in *Escherichia coli *of a streptococcal plasmin receptor J Bacteriol 1992 174 16 5204 5210 1322883 10.1128/jb.174.16.5204-5210.1992 PMC206353

[B6] Pancholi V Fischetti VA A major surface protein on group A streptococci is a glyceraldehyde-3-phosphate-dehydrogenase with multiple binding activity J Exp Med 1992 176 2 415 426 10.1084/jem.176.2.415 1500854 PMC2119316

[B7] Winram SB Lottenberg R The plasmin-binding protein Plr of group A streptococci is identified as glyceraldehyde-3-phosphate dehydrogenase Microbiology 1996 142 2311 2320 10.1099/13500872-142-8-2311 8760943

[B8] Jin H Song YP Boel G Kochar J Pancholi V Group A streptococcal surface GAPDH, SDH, recognizes uPAR/CD87 as its receptor on the human pharyngeal cell and mediates bacterial adherence to host cells J Mol Biol 2005 350 1 27 41 10.1016/j.jmb.2005.04.063 15922359

[B9] Pancholi V Fischetti VA Regulation of the phosphorylation of human pharyngeal cell proteins by group A streptococcal surface dehydrogenase: signal transduction between streptococci and pharyngeal cells J Exp Med 1997 186 10 1633 1643 10.1084/jem.186.10.1633 9362524 PMC2199133

[B10] Terao Y Yamaguchi M Hamada S Kawabata S Multifunctional glyceraldehyde-3-phosphate dehydrogenase of *Streptococcus pyogenes *is essential for evasion from neutrophils J Biol Chem 2006 281 20 14215 14223 10.1074/jbc.M513408200 16565520

[B11] Modun B Williams P The staphylococcal transferrin-binding protein is a cell wall glyceraldehyde-3-phosphate dehydrogenase Infect Immun 1999 67 3 1086 1092 10024547 10.1128/iai.67.3.1086-1092.1999 PMC96433

[B12] Modun B Morrissey J Williams P The staphylococcal transferrin receptor: a glycolytic enzyme with novel functions Trends Microbiol 2000 8 231 237 10.1016/S0966-842X(00)01728-5 10785640

[B13] Seifert KN McArthur WP Bleiweis AS Brady LJ Characterization of group B streptococcal glyceraldehyde-3-phosphate dehydrogenase: surface localization, enzymatic activity, and protein-protein interactions Can J Microbiol 2003 49 5 350 356 10.1139/w03-042 12897829

[B14] Ling E Feldman G Portnoi M Dagan R Overweg K Mulholland F Chalifa-Caspi V Wells J Mizrachi-Nebenzahl Y Glycolytic enzymes associated with the cell surface of *Streptococcus pneumoniae *are antigenic in humans and elicit protective immune responses in the mouse Clin Exp Immunol 2004 138 2 290 298 10.1111/j.1365-2249.2004.02628.x 15498039 PMC1809218

[B15] Schaumburg J Diekmann O Hagendorff P Bergmann S Rohde M Hammerschmidt S Jänsch L Wehland J Kärst U The cell wall subproteome of *Listeria monocytogenes* Proteomics 2004 4 10 2991 3006 10.1002/pmic.200400928 15378750

[B16] Egea L Aguilera L Gimenez R Sorolla MA Aguilar J Badia J Baldoma L Role of secreted glyceraldehyde-3-phosphate dehydrogenase in the infection mechanism of enterohemorrhagic and enteropathogenic *Escherichia coli*: interaction of the extracellular enzyme with human plasminogen and fibrinogen Int J Biochem Cell Biol 2007 39 6 1190 1203 10.1016/j.biocel.2007.03.008 17449317

[B17] Aguilera L Giménez R Badia J Aguilar J Baldoma L NAD+-dependent post-translational modification of *Escherichia coli *glyceraldehyde-3-phosphate dehydrogenase Int Microbiol 2009 12 187 192 19784925

[B18] Alvarez RA Blaylock MW Baseman JB Surface localized glyceraldehyde-3-phosphate dehydrogenase of *Mycoplasma genitalium *binds mucin Mol Microbiol 2003 48 5 1417 1425 10.1046/j.1365-2958.2003.03518.x 12787366

[B19] Kinoshita H Uchida H Kawai Y Kawasaki T Wakahara N Matsuo H Watanabe M Kitazawa H Ohnuma S Miura K Cell surface *Lactobacillus plantarum *LA 318 glyceraldehyde-3-phosphate dehydrogenase (GAPDH) adheres to human colonic mucin J Appl Microbiol 2008 104 1667 1674 10.1111/j.1365-2672.2007.03679.x 18194256

[B20] Ramiah K van Reenen CA Dicks LM Surface-bound proteins of *Lactobacillus plantarum *423 that contribute to adhesion of Caco-2 cells and their role in competitive exclusion and displacement of *Clostridium sporogenes *and *Enterococcus faecalis.* Res Microbiol 2008 159 470 475 10.1016/j.resmic.2008.06.002 18619532

[B21] Nagata H Iwasaki M Maeda K Kuboniwa M Hashino E Toe M Minamino N Kuwahara H Shizukuishi S Identification of the binding domain of *Streptococcus oralis *glyceraldehyde-3-phosphate dehydrogenase for *Porphyromonas gingivalis *major fimbriae Infect Immun 2009 77 5130 5138 10.1128/IAI.00439-09 19737900 PMC2772547

[B22] Gil-Navarro I Gil ML Casanova M O'Connor JE Martinez JP Gozalbo D The glycolytic enzyme glyceraldehyde-3-phosphate dehydrogenase of *Candida albicans *is a surface antigen J Bacteriol 1997 179 16 4992 4999 9260938 10.1128/jb.179.16.4992-4999.1997 PMC179354

[B23] Gozalbo D Gil-Navarro I Azorin I Renau-Piqueras J Martinez JP Gil ML The cell wall-associated glyceraldehyde-3-phosphate dehydrogenase of *Candida albicans *is also a fibronectin and laminin binding protein Infect Immun 1998 66 5 2052 2059 9573088 10.1128/iai.66.5.2052-2059.1998 PMC108162

[B24] Jonathan DC Isla KS Gillian CA Norma RM Neil ARG Nuala AB *Candida albicans *binds human plasminogen: identification of eight plasminogen-binding proteins Mol Microbiol 2003 47 6 1637 1651 10.1046/j.1365-2958.2003.03390.x 12622818

[B25] Lama A Kucknoor A Mundodi V Alderete JF Glyceraldehyde-3-phosphate dehydrogenase is a surface-associated, fibronectin-binding protein of *Trichomonas vaginalis* Infect Immun 2009 77 2703 2711 10.1128/IAI.00157-09 19380472 PMC2708568

[B26] Tettelin H Saunders NJ Heidelberg J Jeffries AC Nelson KE Eisen JA Ketchum KA Hood DW Peden JF Dodson RJ Complete genome sequence of *Neisseria meningitidis *serogroup B strain MC58 Science 2000 287 5459 1809 1815 10.1126/science.287.5459.1809 10710307

[B27] Grifantini R Bartolini E Muzzi A Draghi M Frigimelica E Berger J Ratti G Petracca R Galli G Agnusdei M Previously unrecognized vaccine candidates against group B meningococcus identified by DNA microarrays Nat Biotech 2002 20 9 914 921 10.1038/nbt728 12172557

[B28] Knaust A Weber MV Hammerschmidt S Bergmann S Frosch M Kurzai O Cytosolic proteins contribute to surface plasminogen recruitment of *Neisseria meningitidis* J Bacteriol 2007 189 8 3246 3255 10.1128/JB.01966-06 17307854 PMC1855851

[B29] Tunio SA Oldfield NJ Berry A Ala'Aldeen DAA Wooldridge KG Turner DPJ The moonlighting protein fructose-1, 6-bisphosphate aldolase of *Neisseria meningitidis*: surface localization and role in host cell adhesion Mol Microbiol 2010 76 605 615 10.1111/j.1365-2958.2010.07098.x 20199602

[B30] Kizil G Todd I Atta M Borriello SP Ait-Tahar K Ala'Aldeen DAA Identification and characterization of TspA, a major CD4+ T-cell- and B-cell-stimulating Neisseria-specific antigen Infect Immun 1999 67 3533 3541 10377136 10.1128/iai.67.7.3533-3541.1999 PMC116541

[B31] Hadi HA Wooldridge KG Robinson K Ala'Aldeen DAA Identification and characterization of App: an immunogenic autotransporter protein of *Neisseria meningitidis* Mol Microbiol 2001 41 3 611 623 10.1046/j.1365-2958.2001.02516.x 11532129

[B32] Emanuelsson O Brunak S von Heijne G Nielsen H Locating proteins in the cell using TargetP, SignalP and related tools Nat Protoc 2007 2 4 953 971 10.1038/nprot.2007.131 17446895

[B33] Parkhill J Achtman M James KD Bentley SD Churcher C Klee SR Morelli G Basham D Brown D Chillingworth T Complete DNA sequence of a serogroup A strain of *Neisseria meningitidis *Z2491 Nature 2000 404 6777 502 506 10.1038/35006655 10761919

[B34] Bentley SD Vernikos GS Snyder LAS Churcher C Arrowsmith C Chillingworth T Cronin A Davis PH Holroyd NE Jagels K Meningococcal genetic variation mechanisms viewed through comparative analysis of serogroup C strain FAM18 PLoS Genetics 2007 3 2 e23 10.1371/journal.pgen.0030023 17305430 PMC1797815

[B35] Peng J Yang L Yang F Yang J Yan Y Nie H Zhang X Xiong Z Jiang Y Cheng F Characterization of ST-4821 complex, a unique *Neisseria meningitidis *clone Genomics 2008 91 1 78 87 10.1016/j.ygeno.2007.10.004 18031983

[B36] Pancholi V Chhatwal G Housekeeping enzymes as virulence factors for pathogens Int J Med Microbiol 2003 293 293 391 10.1078/1438-4221-00283 14760970

[B37] Agarwal S Kulshreshtha P Bambah Mukku D Bhatnagar R Alpha-enolase binds to human plasminogen on the surface of *Bacillus anthracis* Biochim Biophys Acta 2008 1784 7-8 986 994 18456007 10.1016/j.bbapap.2008.03.017

[B38] Kim JW Dang CV Multifaceted roles of glycolytic enzymes Trends Biochem Sci 2005 30 3 142 150 10.1016/j.tibs.2005.01.005 15752986

[B39] Jang M Kang HJ Lee SY Chung SJ Sunghyun K Chi SW Cho S Lee S Lee CK Par BC Glyceraldehyde-3-phosphate, a glycolytic intermediate, plays a key role in controlling cell fate via inhibition of caspase activity Mol Cells 2009 28 559 563 10.1007/s10059-009-0151-7 19937139

[B40] Read RC Zimmerli S Broaddus C Sanan DA Stephens DS Ernst JD The (alpha2-->8)-linked polysialic acid capsule of group B *Neisseria meningitidis *modifies multiple steps during interaction with human macrophages Infect Immun 1996 64 8 3210 3217 8757855 10.1128/iai.64.8.3210-3217.1996 PMC174209

[B41] Stephens DS Spellman PA Swartley JS Effect of the (alpha 2-->8)-linked polysialic acid capsule on adherence of *Neisseria meningitidis *to human mucosal cells J Infect Dis 1993 167 2 475 479 8380612 10.1093/infdis/167.2.475

[B42] Saad N Urdaci M Vignoles C Chaignepain S Tallon R Schmitter JM Bressollier P *Lactobacillus plantarum *299v surface-bound GAPDH: a new insight into enzyme cell walls location J Microbiol Biotechnol 2009 19 1635 1643 10.4014/jmb.0902.0102 20075631

[B43] van Vliet AH Wooldridge KG Ketley JM Iron-responsive gene regulation in a *Campylobacter jejuni fur *mutant J Bacteriol 1998 180 5291 5298 9765558 10.1128/jb.180.20.5291-5298.1998 PMC107575

