# Supplementary material for: The role of glyceraldehyde 3-phosphate dehydrogenase (GapA-1) in Neisseria meningitidis adherence to human cells
Source: BMC Microbiol. 2010 Nov 9;10:280. doi: 10.1186/1471-2180-10-280 (PMC2994834; doi:10.1186/1471-2180-10-280)
Supplement: Additional file 1 — Isolates of N. meningitidis examined for the expression of GapA-1. [file 1471-2180-10-280-S1.DOC]

**Table 2 - Isolates of *N. meningitidis*** examined for the expression of GapA-1

| Strain* | Country of origin | Date of isolation | Disease | Serogroup | Sequence type | Clonal complex |
| --- | --- | --- | --- | --- | --- | --- |
| Z1035 | Pakistan | 1967 | meningitis and septicaemia | A | 1 | ST-1 complex/subgroup I/II |
| Z1054 | Finland | 1975 | invasive (unspecified) | A | 5 | ST-5 complex/subgroup III |
| Z1213 | Ghana | 1973 | invasive (unspecified) | A | 4 | ST-4 complex/subgroup IV |
| Z1534 | UK | 1941 | invasive (unspecified) | A | 21 | unknown |
| Z3771 | UK | 1987 | invasive (unspecified) | A | 5 | ST-5 complex/subgroup III |
| Z3842 | Norway | 1976 | invasive (unspecified) | B | 32 | ST-32 complex/ET-5 complex |
| Z4181 | Mali | 1989 | carrier | C | 11 | ST-11 complex/ET-37 complex |
| Z4667 | Netherlands | 1963 | invasive (unspecified) | B | 48 | ST-41/44 complex/Lineage 3 |
| Z4673 | Netherlands | 1986 | invasive (unspecified) | B | 41 | ST-41/44 complex/Lineage 3 |
| Z4676 | Denmark | 1962 | invasive (unspecified) | B | 37 | ST-37 complex |
| Z4678 | East Germany | 1985 | invasive (unspecified) | B | 19 | ST-18 complex |
| Z6413 | South Africa | 1990 | invasive (unspecified) | C | 8 | ST-8 complex/Cluster A4 |
| Z6414 | New Zealand | 1994 | invasive (unspecified) | C | 66 | ST-8 complex/Cluster A4 |
| Z6420 | Greece | 1992 | invasive (unspecified) | B | 41 | ST-41/44 complex/Lineage 3 |

*Further details of strains are available at http://pubmlst.org/
